# Geosocial Networking Smartphone App Use and High-Risk Sexual Behaviors Among Men Who Have Sex With Men Attending University in China: Cross-sectional Study

**DOI:** 10.2196/31033

**Published:** 2022-03-28

**Authors:** Song Fan, Peiyang Li, Yuqing Hu, Hui Gong, Maohe Yu, Yi Ding, Zhenzhou Luo, Guohui Wu, Lin Ouyang, Huachun Zou

**Affiliations:** 1 School of Public Health Southwest Medical University Luzhou China; 2 School of Public Health Sun Yat-sen University Guangzhou China; 3 School of Public Health (Shenzhen) Sun Yat-sen University Shenzhen China; 4 Tianjin Center for Disease Control and Prevention Tianjin China; 5 Nanshan Center for Chronic Disease Control Shenzhen China; 6 Chongqing Center for Disease Control and Prevention Chongqing China; 7 Kirby Institute University of New South Wales Sydney Australia; 8 School of Public Health Shanghai Jiao Tong University Shanghai China

**Keywords:** gay app, men who have sex with men, student, China, smartphone, mobile phone

## Abstract

**Background:**

Gay apps are smartphone-based geosocial networking apps where many men who have sex with men (MSM) socialize and seek sex partners. Existing studies showed that gay app use is associated with greater odds of high-risk sexual behaviors and potentially more HIV infections. However, little is known about this behavior among young MSM.

**Objective:**

We conducted this study to understand gay app use and its influencing factors among MSM attending university in China.

**Methods:**

From January to March 2019, participants were recruited from 4 regions with large populations of college students in China: Chongqing, Guangdong, Shandong, and Tianjin. The eligibility criteria were MSM aged 16 years or older, self-identified as a university student, and being HIV negative. A self-administered online structured questionnaire was used to collect data on sociodemographic information, sexual behaviors, gay app use, substance use, and HIV testing history. We performed multivariable log-binomial regression to assess correlates of seeking sex partners via gay apps.

**Results:**

A total of 447 MSM attending university with an average age of 20.4 (SD 1.5) years were recruited. Almost all participants (439/447, 98.2%) reported gay app use at some point in their life, and 240/439 (53.7%) reported ever seeking sex partners via gay apps. Blued (428/439, 97.5%) was the most popular gay app. Higher proportions of sexual risk behaviors (including seeking sex partners via apps [*P*<.001], engaging in group sex [*P*<.001], having multiple sex partners [*P*<.001], unawareness of sex partners’ HIV status [*P*<.001], and using recreational drugs during sex [*P*<.02]) were positively associated with the increase in the frequency of gay app use. In multivariable analysis, participants who used gay apps to seek sex partners might be more likely to have multiple sex partners in the past 3 months (adjusted prevalence ratio [APR] 1.53, 95% CI 1.33-1.76; *P*<.001), engage in group sex in the past 3 months (APR 1.55, 95% CI 1.35-1.78; *P*<.001), and have sex partners with unknown or positive HIV status (APR 1.72, 95% CI 1.46-2.01; *P*<.001).

**Conclusions:**

Seeking sex partners via gay apps may associate with the increased high-risk sexual behaviors among MSM attending university. The causality between seeking sex partners via gay apps and increased high-risk sexual behaviors should be further investigated so as to inform potential policies for HIV prevention.

**Trial Registration:**

Chinese Clinical Trial Registry ChiCTR1900020645; http://www.chictr.org.cn/showprojen.aspx?proj=34741

## Introduction

Gay apps are smartphone-based geosocial networking apps where men who have sex with men (MSM) socialize. In recent years, gay apps have gained increasing popularity in the MSM community. Grindr has more than 3 million daily users in nearly 200 countries [1,2]. Blued is the first gay app originating from China and has more than 40 million registered users worldwide [3]. Gay apps have become the main platform for seeking sex partners and romantic relationships among MSM [4,5] thanks to the geo-positioning function, which facilitates MSM to socialize with nearby partners [5,6].

The widespread use of gay apps brings about challenges to HIV prevention in MSM. Existing studies showed that gay app users reported more sex partners than nongay app users [7,8]. Gay app users had more frequent sexual behaviors and were more likely to engage in unprotected sex with partners met via gay apps [8,9]. Moreover, MSM who have used gay apps for over 1 year were more likely to drink alcohol, use illicit drugs, and abuse substances, making them more vulnerable to HIV [10]. However, some studies found gay apps might be conducive to HIV control, as gay apps could be used to disseminate HIV knowledge [11] and promote HIV testing [12].

MSM attending university in China have become a key population of HIV/AIDS transmission [13,14]. The number of annual newly diagnosed HIV cases among students is over 3000 since 2017, which is mostly attributable to homosexual behavior among men [15]. High-risk behaviors such as condomless sex, group sex, and drug use during anal intercourse are prevalent among MSM attending university, and these behaviors may render this population more vulnerable to HIV [16]. Compared with nonstudent MSM, it is more difficult to approach and investigate this young group. This is largely because most of them only recently realized their sexual orientation and are not ready to disclose their sexuality. They are also concerned about stigma from peers if they disclose their sexuality [17]. Their discrete status is a barrier to timely access to HIV prevention and care services.

Previous studies have found that MSM using gay apps tend to be younger and better educated [1], raising concerns about potential effects of gay app use on MSM attending university. However, literature on gay app use in MSM attending university is scarce and the following aspects are not well understood: (1) the characteristics of MSM attending university who use gay apps. (2) Does the use of gay apps facilitate risky sex behaviors, thus leading to an increased risk of HIV infection? (3) Can gay apps be utilized to promote sexual health among this young population? Therefore, this study aims to understand (1) the relationship between frequency of gap app use and engagement in sexual risk behaviors, (2) the relationship between seeking sex partners via gay apps and engagement in sexual risk behaviors.

## Methods

### Participants

From January to April 2019, the study was conducted in 4 regions with large populations of college students in China, including Chongqing in southwest China, Guangdong in south China, Shandong in east China, and Tianjin in north China. Individuals were eligible for participation if they were male; aged 16 years or older; university student (technical diploma and undergraduate students); MSM (having sexual behaviors including mutual masturbation, oral sex, or anal sex with male partners); HIV negative or unknown; and willing to provide informed consent. Each participant received free HIV testing services and 50 Chinese Yuan (~US $7.9) as compensation after enrollment. All data were stored anonymously in encrypted storage space, and personally identifiable information was stored separately from other behavioral and test result data.

### Ethical Approval

The study has been approved by the Ethics Review Board of Sun Yat-sen University (SYSU-SPH2018044) and registered with the Chinese Clinical Trial Registry (Registration number: ChiCTR1900020645).

### Procedure and Measurements

For sampling and recruitment, the methodology has previously been published elsewhere [18]. Briefly, recruitment of participants was promoted both online and offline. For online recruitment, study advertising campaigns were placed on the social network. For offline recruitment, the study advertisement was distributed to the collaboration of community-based organizations (CBOs), health clinics, and student associations. After reading study advertisements online or offline, participants could make an appointment to join the study at a preferred CBO. After scanning the QR code on a smartphone, participants finished the online questionnaire at the CBO site. A self-administrated online structured questionnaire was used to collect each participant’s sociodemographic information (including age, ethnicity, educational status, involvement in class leadership, academic performance, student loans, sexual orientation, and gender of sex partner in lifetime), sexual behaviors (including the age at first anal intercourse, commercial sex in lifetime, forced sex in lifetime, group sex in the past 3 months, number of sex partners in the past 3 months, condomless sex in the past 3 months, and HIV serostatus of sex partners in the past 3 months), gay app use, recreational drug use, and HIV testing. Gay app use was assessed by the question, “Have you ever used any gay app?”, and subsequent questions about specific gay app use behaviors, including preference, duration, frequency, and purpose. We also assessed seeking sex partners via gay apps by the question “During the past 3 months, have you ever used gay apps to seek sex partners?”, and subsequent questions including the number of sex partners, group sex, condomless sex, and HIV serostatus of sex partners. Recreational drug use was assessed by asking “Have you ever used any of the below-listed recreational drugs during sex? Popper (alkyl nitrites), ecstasy, crystal methamphetamine, and ketamine.” The HIV testing was conducted with rapid HIV testing (Alere Determine HIV-1/2; Alere Medical Co., Ltd.) at the location of CBOs; those who tested positive were referred to the local Centers for Disease Control and Prevention (CDC) for confirmation testing and subsequent treatment services [19]. HIV infections in this study were confirmed results.

### Statistical Analysis

We used mean and SD to describe continuous variables, and proportions to describe categorical variables. Chi-square test for trend was used to test the linear trend in the proportion of engaging in sexual risk behaviors with the increase in the frequency of app use. Univariable log-binomial regressions were performed to assess correlates (sociodemographic variables, sexual behaviors) of seeking sex partners via gay apps. Multivariable log-binomial regressions were conducted to use *seeking sex partners via the gay apps* as the independent variable and *sexual behavior in past 3 months* (including group sex, number of sex partners, condomless sex, and HIV status of sex partners) as the dependent variable. Each variable of sexual behaviors was examined independently in separate regression models, adjusted for sociodemographic variables. Variables with *P*<.05 were retained in the final model. Results were presented with an adjusted prevalence ratio (APR) and 95% CI. All data were analyzed using R (version 3.6.1; R Foundation).

## Results

### Participants

A total of 447 MSM attending university with an average age of 20.4 (SD 1.5) years were recruited in the study (Table 1). The majority of participants self-identified as gay (382/447, 85.5%). Most participants were undergraduate (355/447, 79.4%) students and self-reported academic performance above the average (354/447, 79.2%).

**Table 1:** Characteristics and gay app use among men who have sex with men attending university in China (N=447)

Variables		Values, n (%)
**Age (years)**		
	≤20	236 (52.8)
	≥21	211 (47.2)
**Education**		
	Undergraduate	355 (79.4)
	Junior college	92 (20.6)
**Academic performance**		
	Below average	93 (20.8)
	Average and above	354 (79.2)
**Sexual orientation**		
	Homosexual	382 (85.5)
	Bisexual	44 (9.8)
	Other	21 (4.7)
**Gay apps used^a^**		
	Blued	428 (97.5)
	Aloha	226 (51.5)
	Jack’d	38 (8.7)
	Grindr	37 (8.4)
	Others	33 (7.5)
**The most frequently used apps (past 3 months; n=439)**		
	Blued	318 (72.4)
	Aloha	84 (19.1)
	Others	37 (8.4)
**Number of gay apps used (n=439)**		
	1	393 (89.5)
	≥2	46 (10.5)
**Length of gay app use (months; n=439)**		
	≤5	55 (12.5)
	6-12	30 (6.8)
	≥13	354 (80.6)
**Frequency of gay app use (past 3 months; n=439)**		
	Monthly	125 (28.5)
	Weekly	173 (39.4)
	Daily	141 (32.1)
**Time (hours) spent on gay app per day (past 3 months; n=439)**		
	≤0.5	362 (82.5)
	1	38 (8.7)
	≥2	39 (8.9)
**Purpose of gay app use (past 3 months; n=439)^a^**		
	Relationships	199 (45.3)
	Following others’ updates	185 (42.1)
	Making friends	171 (39.0)
	Hooking up with sex partners	161 (36.7)
	Chatting	119 (27.1)
	Watching live streaming	56 (12.8)
**Type of sexual behavior with sex partners known via gay apps (past 3 months; n=240)^a^**		
	Anal sex	197 (82.1)
	Oral sex	164 (68.3)
	Masturbation	97 (40.4)
**HIV status of last sex partners known via gay apps (past 3 months; n=240)**		
	Negative	93 (38.8)
	Positive	6 (2.5)
	Unknown	141 (58.8)

^a^Multiple choice.

### Gay App Use

Among MSM attending university enrolled in the study, 439/447 (98.2%) reported ever using gay apps (Table 1). The most popular gay apps were Blued (428/439, 97.5%) and Aloha (226/439, 51.5%); likewise, the most frequently used gay apps in the past 3 months were also Blued (318/439, 72.4%) and Aloha (84/439, 19.1%). Most participants reported using only 1 type of app (393/439, 89.5%), over 12 months of usage time (354/439, 80.6%), and using less than 1 hour per day (362/439, 82.5%). The top 3 purposes for using gay apps were looking for a romantic relationship (199/439, 45.3%), following others’ updates (185/439, 42.1%), and making friends (171/439, 39.0%). The majority of participants (197/240, 82.1%) reported engaging in anal sex with sex partners met via apps and over half (141/240, 58.8%) did not know the HIV status of last sex partner.

**Figure 1:**
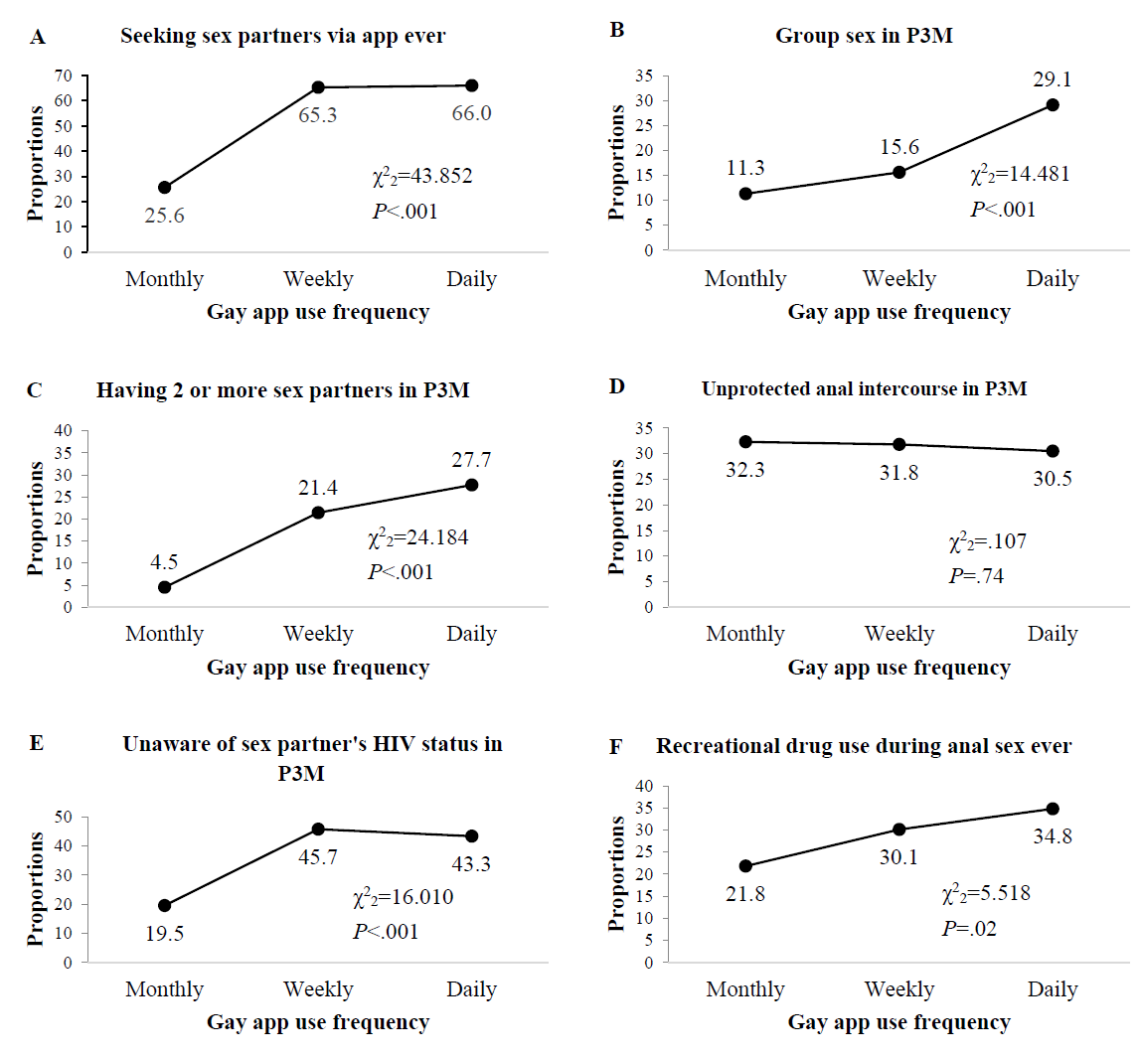
Behavioral changes over increasing frequency of gay app use among MSM attending university: (A) seeking sex partners with gay apps use frequency; (B) group sex with gay apps use frequency in past 3 months; (C) having two or more sex partners with gay apps use frequency in past 3 months; (D) unprotected anal intercourse with gay apps use frequency in past 3 months; (E) unaware of sex partners’ HIV status with gay apps use frequency in past 3 months; (F) recreational drug use during anal sex ever. P3M: Past 3 months.

The frequencies of gay app use were months (125/439, 28.5%), weeks (173/439, 39.4%), and days (141/439, 32.1%) in the past 3 months among MSM attending university (Table 1). Chi-square test for trend showed that higher proportions of sexual behaviors, including seeking sex partners via apps (*P*<.001), engaging in group sex (*P*<.001), having multiple sex partners (*P*<.001), unknown sex partners’ HIV status (*P*<.001), and using recreational drugs during sex (*P*<.02), were positively associated with the increase in the frequency of gay app use (Figure 1).

### Seeking Sex Partner via Gay Apps

Table 2 shows the characteristics of seeking sex partners via gay apps among MSM attending university. As many as 240 reported using gay apps to seek sex partners. In multivariable analysis, participants who used gay apps to seek sex partners were more likely to have multiple sex partners in the past 3 months (APR 1.53, 95% CI 1.33-1.76, *P*<.001), engage in group sex in the past 3 months (APR 1.55, 95% CI 1.35-1.78, *P*<.001), and have sex partners with unknown or positive HIV status (APR 1.72, 95% CI 1.46-2.01, *P*<.001).

**Table 2:** Correlates of seeking sex partner via gay apps among men who have sex with men attending university in China for past 3 months.

Variables		N		Seeking sex partner via gay apps				
				n (%)	Univariate prevalence ratio (95% CI)		Adjusted prevalence ratio (95% CI)	
**Age (years)**								
	≤20	236	122 (51.7)			1		
	≥21	211	118 (55.9)			1.08 (0.91-1.29)		
**Education**								
	Undergraduate	355	193 (54.4)			1		
	Junior college	92	47 (51.1)			0.94 (0.75-1.17)		
**Academic performance**								
	Below average	93	48 (51.6)			1		
	Average and above	354	192 (54.2)			0.95 (0.77-1.18)		
**Sexual orientation**								
	Other	21	10 (47.6)			1		
	Bisexual	44	23 (52.3)			1.21 (0.43-3.41)		
	Homosexual	382	207 (54.2)			1.30 (0.54-3.14)		
**Gender of sex partner**								
	Only male	423	231 (54.6)			1		
	Both male and female	13	6 (46.2)			1.18 (0.65-2.14)		
**Age at first anal intercourse (years)**								
	≤17	113	65 (57.5)			1		
	≥18	323	172 (53.3)			1.08 (0.90-1.30)		
**Commercial sex (lifetime)**								
	No	420	220 (52.4)			1		
	Yes	27	20 (74.1)			1.84 (0.96-3.50)		
**Forced sex (lifetime)**								
	No	418	231 (55.3)			1		
	Yes	18	9 (50.0)			0.92 (0.57-1.47)		
**Group sex (past 3 months)**								
	No	364	170 (46.7)			1		1
	Yes	83	70 (84.3)			3.40 (2.05-5.66)		1.55 (1.35-1.78)
**Number of sex partners (past 3 months)**								
	≤2	365	166 (45.5)			1		1
	≥3	82	74 (90.2)			5.59 (2.87-10.87)		1.53 (1.33-1.76)
**Condomless sex (past 3 months)**								
	No	306	144 (47.1)			1		1
	Yes	141	96 (68.1)			1.24 (0.98-1.57)		0.98 (0.84-1.14)
**HIV status of sex partners (past 3 months)**								
	Negative	281	111 (39.5)			1		1
	Positive or unknown	166	129 (77.7)			1.97 (1.67-2.32)		1.72 (1.46-2.01)
**Recreational drug use^a^ during sex**								
	No	317	154 (48.6)			1		
	Yes	130	86 (66.2)			1.52 (1.17-1.98)		
**HIV testing**								
	No	49	25 (51.0)			1		
	Yes	398	215 (54.0)			1.07 (0.79-1.45)		
**HIV infection**								
	Negative	437	234 (53.5)			1		
	Positive	10	6 (60.0)			1.16 (0.54-2.50)		

^a^Recreational drug includes popper (alkyl nitrites), ecstasy, crystal methamphetamine, and ketamine.

## Discussion

### Principal Findings

To our knowledge, this is the first study to characterize gay app use among MSM attending university in China. We found that almost all MSM attending university used gay apps, and those who used gay apps to seek sexual partners were likely to be more engaging in high-risk sexual behaviors, including multiple sexual partners, group sex, commercial sex, and recreational drug use during sex.

We found a high prevalence (439/447, 98.2%) of gay app use among MSM attending university, which was much higher than that in nonstudent MSM (ranging from 40% [20] to 60% [21,22]). Previous studies have shown the differences in age distribution for app use among MSM, with younger age groups using them more frequently [21,23]. The mean age of participants in our study was 20 (SD 1.5) years, indicating that MSM attending university were a younger subgroup of MSM. Therefore, they are more likely to use gay apps compared with nonstudent MSM.

The popularity of gay app use among MSM attending universities was accompanied by some high-risk behaviors. Our study found that high-risk behaviors, including engaging in group sex in the past 3 months, having 2 or more sex partners in the past 3 months, failing to know sex partners’ HIV status in the past 3 months, and using recreational drugs during sex, increased with the frequency of gay app use. Previous studies have indicated that men using gay apps could facilitate sexually risky behaviors [7,22,24]. Moreover, MSM attending university who used gay apps to seek sex partners were more likely to have multiple sex partners, engage in group sex and commercial sex, and use recreational drugs. This is consistent with previous studies among nonstudent MSM in China that found gay app users had more sex partners and more group sex compared with those who did not use apps to seek sex partners [20,22,25]. Notably, high-risk behaviors, including multiple sex partners, group sex, and commercial sex, could increase the risk of infection with HIV or other sexually transmitted infections. Therefore, additional interventions are needed to address the sexual risk behaviors associated with seeking sex partners via apps.

Literally all MSM attending university have used gay apps at some point, which points to the potential feasibility of using gay app–based platforms to engage them in HIV prevention and care programs. Barriers, such as stigma, discrimination, and disclosure of sexuality, prevent young MSM from seeking traditional venue–based HIV prevention services. Because social networking apps are very popular among young people [26,27], there may be opportunities to facilitate HIV prevention efforts via these apps. Some app-based HIV interventions achieved desirable results. For example, since an HIV testing campaign using app push messages to promote HIV testing has been launched on Blued in 2015, over 15,000 MSM have tested for HIV, of whom over 2000 self-identified as university students. The number of those who tested for HIV increased substantially from 836 in 2013 before the campaign to 7315 in 2017 after the campaign [12]. Given that gay apps can instantly reach millions of targeted individuals, app-based interventions include information on HIV self-testing, pre-exposure prophylaxis, post-exposure prophylaxis, and HIV treatment as prevention, and thus may benefit a broad group in the MSM community.

We found Blued and Aloha were the most popular gay apps among MSM attending university, and the majority of those reported using only 1 app (393/439, 89.5%). There were 43 brands of gay apps in China in 2018 [28], and among nonstudent MSM 65.9% used only 1 app, and 23.2% used 2 apps [21]. Currently, there are regional differences in gay app use. While English language–based gay apps such as Grindr and Jack’d have subscribers from many countries, the gay app market in China is dominated by local apps such as Blued and Aloha. Those differences in gay app choices may be attributed to linguistic and cultural differences among MSM. Blued and Aloha are also platforms for HIV care services.

### Limitations

There were limitations. First, there may be a selection bias in the study population. Participants were recruited mainly through online advertisements and enrolled offline for HIV testing at CBO site, which may exclude student MSM who hide their sexual orientation and were not willing to attend on-site testing at CBO. This deeply hidden population may be frequent users of gay apps, and the lack of such a population among participants may limit the generalizability of the study. Second, the study results may be affected by social desirability reporting bias because sensitive private information was based on self-reporting by participants. Third, the design of the cross-sectional study prevents making causal inferences between using an app to seek sex partners and risk behaviors.

### Conclusions

Our study suggests high gay app use among MSM attending university and using apps to seek sex partners may be associated with increased high-risk sexual behaviors. The causality between seeking sex partners via gay apps and increased high-risk sexual behaviors should be further investigated so as to inform potential policies for HIV prevention.

## Abbreviations

APR: adjusted prevalence ratio
CBO: community-based organization
CDC: Centers for Disease Control and Prevention
MSM: men who have sex with men
